# Subjective Sound Quality Detection (HISQUI) over Time after Vibrant Soundbridge Implantation

**DOI:** 10.3390/jcm11071811

**Published:** 2022-03-25

**Authors:** Christof Buhl, Valeria Schindler, Flurin Pfiffner, Dorothe Veraguth, Alexander Huber, Christof Röösli

**Affiliations:** 1 Department of Otorhinolaryngology-Head and Neck Surgery, University Hospital Zurich, University of Zurich, 8091 Zurich, Switzerland; christof.buhl@luks.ch (C.B.); flurin.pfiffner@usz.ch (F.P.); dorothe.veraguth@usz.ch (D.V.); alex.huber@usz.ch (A.H.); 2 Department of Gastroenterology, University Hospital Zurich, University of Zurich, 8091 Zurich, Switzerland; valeria.schindler@usz.ch

**Keywords:** Vibrant Soundbridge, long term follow up, free-field audiogram, HISQUI, speech in noise test

## Abstract

Background: To evaluate the long-term audiological outcomes combined with the Hearing Implant Sound Quality Index (HISQUI) after Vibrant Soundbridge (VSB) implantation. Methods: Prospective recall cohort study of patients who received a VSB in a tertiary academic medical center between 1996 and 2017. Air conduction (AC) and bone conduction (BC), sound field thresholds in aided and unaided conditions, and speech discrimination in noise (Oldenburger sentence test) were measured. Postoperative results were compared with preoperative audiograms. Furthermore, the HISQUI was evaluated. Results: Ten patients (eleven implants) were included, the mean follow up period was nine years. The mean AC threshold preoperatively was between 63 and 70 dB, and the BC was between 38 and 49 dB from 500 to 4000 Hz. In the free-field audiogram, the mean threshold was between 61 and 77 dB unaided vs. between 28 and 52 dB in the aided condition. The average signal to noise ratio (SNR) in the Oldenburger sentence test in the unaided condition was 10 dB ± 6.7 dB vs. 2 dB ± 5.4 dB in the aided condition. Three patients reported a good to very good hearing result, four patients a moderate, and three patients a poor hearing result. There was a significant association between the years of implantation and the HISQUI (*p* = 0.013), as well as a significant decrease by 14 HISQUI points per 10 dB SPL decline (SE 5.2, *p* = 0.023). There was a significant difference between the change of BC over the years and the HISQUI, as well as the number of years after implantation and the HISQUI. On average, per dB decrease in BC, the HISQUI decreases by 1.4 points, and every year after implantation the HISQUI decreases by 2.7 points. Conclusions: The aided threshold in free field and speech understanding in noise improved significantly with VSB. An increase over time of BC thresholds was observed as well as a decrease in HISQUI score. This decrease in BC thresholds over time may be due to presbycusis. Therefore, monitoring of these patients over time should be considered to discuss alternative hearing rehabilitation measures in a timely manner.

## 1. Introduction

It has been shown that an active middle ear implant is an alternative for patients with conductive or mixed hearing loss, who do not benefit from conventional hearing aids or cannot wear a conventional hearing aid because of aural atresia, chronic inflammations of the outer ear canal, severe allergies, or inflammations arising from wearing hearing aids. One commercially available active middle ear implant is the Vibrant Soundbridge (VSB) (Med-El, Innsbruck, Austria). The VSB consists of two primary components: the audio processor (AP) as an external unit, and the surgically implanted vibrating ossicular prosthesis (VORP). The AP consists of a microphone, signal processing electronics, a telemetry coil, and a battery to receive and process incoming acoustical signals transdermal to the implanted VORP. The VORP consists of three further components: the implanted receiver unit, the conductor link, and the Floating Mass Transducer (FMT) [1,2]. 

The first clinical trials to investigate results after VSB implantation in Europe and the United States started in 1996 [3]. Since then, approximately 40 patients have been implanted in our department. The VSB has become a well-established active middle ear implant and it is reported to be efficient and safe for the rehabilitation of conductive and mixed hearing loss when conventional hearing aids cannot be used [4,5]. 

There is no standardized questionnaire to evaluate patient satisfaction for active middle ear implants. For cochlear implant users, the Hearing Implant Sound Quality Index (HISQUI19) was developed and validated to evaluate the self-perceived level of auditory benefit in cochlear implant patients. The questionnaire consists of 19 questions describing every day listening situations [6]. As a user-friendly questionnaire, other authors have also used this tool to determine the subjective auditory benefit in other implants, such as the Bonebridge hearing implant [7]. 

There are relatively few studies reporting on the audiological outcomes together with subjective benefits for VSB [8]. Therefore, this study aims to analyze the audiometric evaluation with a follow-up of up to 20 years, as well as to report on patients’ subjective hearing benefit. We hypothesized that the VSB is a reliable device and that patients show a significant benefit.

## 2. Materials and Methods

The study protocol was approved by the local Ethical Committee (KEK-ZH No. 2016-01593). All patients received an implantation of an active middle ear implant (Soundbridge) between 1996 and 2017 at the ENT Department, University Hospital of Zurich, Switzerland. All study participants were ≥18 years old and provided written informed consent prior to the examinations. 

### 2.1. Pure-Tone Audiogram

All audiometric tests were performed following standard procedures in accordance with ISO 8253-1. An AC pure-tone audiogram for each side with headphones was conducted for 500, 1000, 2000, 3000, and 4000 Hz at two time points: (1) before surgery, (2) at the time of follow up with the middle ear implant device turned off. The results were compared to detect changes in the hearing threshold. At the time of follow-up, a free field audiometry test was performed with the same frequencies with the stimulus coming from a loudspeaker 1 m in the front of the participants. First, the active middle ear implant was turned off (unaided), then we performed the test with the device turned on (aided). To rate the benefit of the middle ear implant, the two tests were compared. The hearing tests were measured with appropriate contralateral masking through calibrated headphones.

### 2.2. Speech Discrimination

Speech discrimination was evaluated using the Oldenburger sentence test in noise (OLSA) [9]. The OLSA-noise (pseudo continuous) served as the noise source and was located in the frontal position (N). One long sentence list was used to measure each situation. The sound signal was presented from the front. The noise level was set at 65 dB SPL, and the speech level was changed adaptively according to the number of words repeated correctly. Results were measured as a signal-to-noise ratio (SNR) in dB, indicating the difference in the sound signal level at which 50% of the words were repeated correctly with a constant noise level of 65 dB SPL. The SNRs were measured once with the implant turned on as the aided condition and once with the implant turned off as the unaided condition. Thus, the ‘‘benefit’’ was defined as a nominal decrease in the SNR, expressed as a negative value, calculated as the difference in the SNR between the aided and unaided condition. The non-tested ear was occluded with an earplug inserted deeply into the ear canal.

### 2.3. Questionnaires

To evaluate the subjective hearing benefit of the implant, the HISQUI19 questionnaire was used [6]. This index is often used to evaluate the subjective hearing benefit in patients with CI implantation or in patients with bimodal hearing devices (CI and hearing aids) [10]. This questionnaire consists of 19 questions on a 7-point Likert scale rating the self-perceived level of auditory benefit. The achieved total score corresponds to 1 of 5 categories (<30 = very poor sound quality up to ≥111 = very good sound quality). Questions include: “can you effortlessly distinguish between a male and a female voice?”, or “when talking on the phone, can you effortlessly understand the voices of familiar people?”. 

### 2.4. Statistical Analysis

Statistical analysis was performed with R version 3.2.0 (R Foundation for Statistical Computing, Vienna, Austria). Graphs and figures were constructed with R and GraphPad Prism (8.3.1). Data of pure tone audiograms, as well as free-field audiogram measurements and signal-to-noise ratios, were reported as mean ± standard deviation (SD). Linear regression was used to test the associations between the subjective hearing benefit and the audiometric parameters of inner ear function. The level of statistical significance was set at *p* < 0.05. 

## 3. Results

### 3.1. Demographics

Thirty-seven patients have been implanted with VSB in our tertiary academic hospital center between 1996 and 2017. Ten patients could not be contacted because they moved away without leaving their contact data, nine refused further tests, five were under 18 years old or not able to give informed consent due to their mental state, two did not use the implant anymore, and one patient had passed away (dropout rate of 73%). Ten implanted patients (four female, six male) were included in this study. The demographics of included patients are shown in Table 1. 

### 3.2. Pure Tone Audiogram 

**Figure 1:**
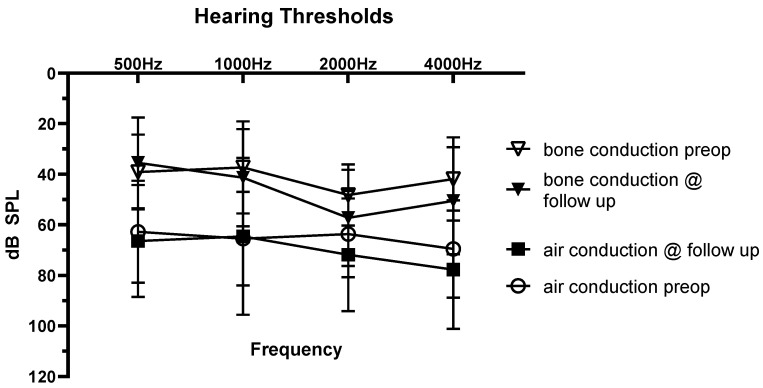
Pre- and postoperative AC and BC hearing thresholds in pure tone audiogram (mean and SD).

The average AC threshold preoperatively was 63 dB ± 20 dB SPL at 500 Hz, 65 dB ± 19 dB SPL at 1000 Hz, 64 dB ± 17 dB SPL at 2000 Hz, and 70 dB ± 19 dB SPL at 4000 Hz. The average BC threshold preoperatively was 39 dB ± 15 dB SPL at 500 Hz, 38 dB ± 19 dB SPL at 1000 Hz, 49 dB ± 14 dB at 2000 Hz, and 42 dB ± 16 dB at 4000 Hz (Figure 1). 

The average unaided AC threshold postoperatively was 66 dB ± 22 dB SPL at 500 Hz, 70 dB ± 24 dB SPL at 1000 Hz, 72 dB ± 22 dB SPL at 2000 Hz, and 77 dB ± 22 dB SPL at 4000 Hz. The average BC threshold postoperatively was 35 dB ± 18 dB SPL at 500 Hz, 41 dB ± 19 dB SPL at 1000 Hz, 57 dB ± 19 dB at 2000 Hz, and 50 dB ± 21 dB at 4000 Hz. Pre- and postoperatively, hearing thresholds did not change significantly. For AC, they were 3 dB for 500 Hz, 5 dB for 1000 Hz, 8 dB for 2000 Hz, and 7 dB for 4000 Hz. For BC, the difference was −dB for 500 Hz, 3 dB for 1000 Hz, 8 dB for 2000 Hz, and 8 dB for 4000 Hz (Figure 1).

### 3.3. Free-Field Audiogram 

**Figure 2:**
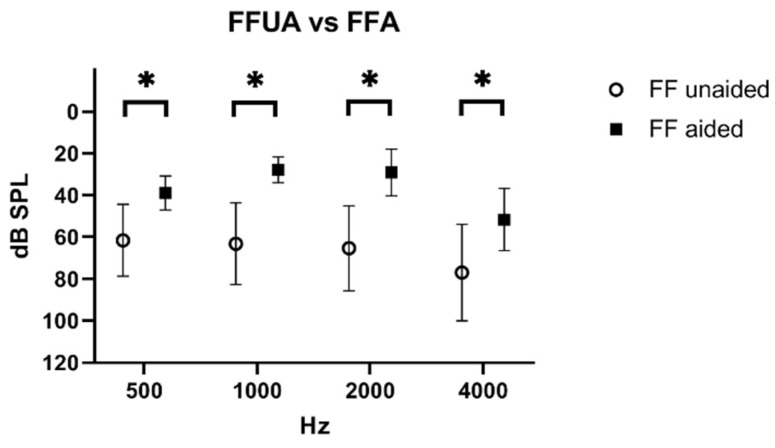
Free-field with a turned off (unaided) device compared to free-field with a turned on (aided) device (mean and SD). Significant differences are indicated with *.

The average threshold with the device turned off (unaided) in the free-field audiogram was 61 dB ± 17 dB SPL at 500 Hz, 63 dB ± 19.5 dB at 1000 Hz, 65 dB ± 20 dB at 2000 Hz, and 77 dB ± 23 dB at 4000 Hz. The average threshold with the device turned on (aided) was significantly lower: 39 dB ± 8 dB at 500 Hz (*p* = 0.008), 28 dB ± 6 dB at 1000 Hz (*p* = 0.002), 29 dB ± 11 dB at 2000 Hz (*p* = 0.003), and 52 ± 15 dB at 4000 Hz (*p* = 0.034). The average gain in dB SPL with the device active (aided) in the free-field audiometry was 23 dB ± 17 dB SPL at 500 Hz, 35 dB ± 17 dB at 1000 Hz, 36 dB ± 24 dB at 2000 Hz, and 25 dB ± 26 dB at 4000 Hz (Figure 2). 

**Figure 3:**
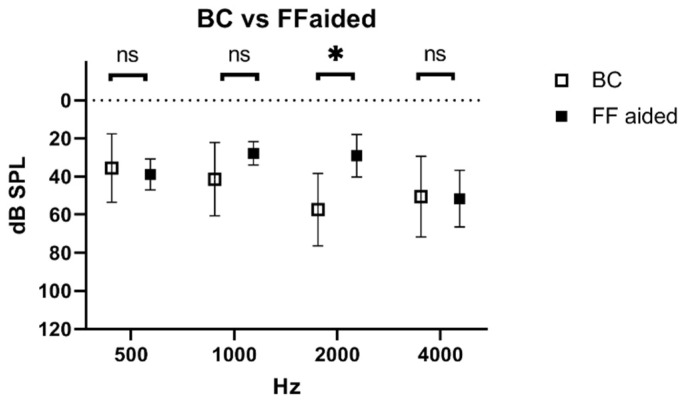
BC compared to free-field with the device turned on (aided) (mean and SD). Significant differences are indicated with *, not significant differences are indicated as “ns”.

The difference between BC postoperatively and the free-field audiogram with the device turned on (aided) was 28 dB in 2000 Hz (*p* < 0.01) and showed no difference in 500 Hz, 1000 Hz, and 4000 Hz (Figure 3).

### 3.4. Speech Discrimination in Noise

**Figure 4:**
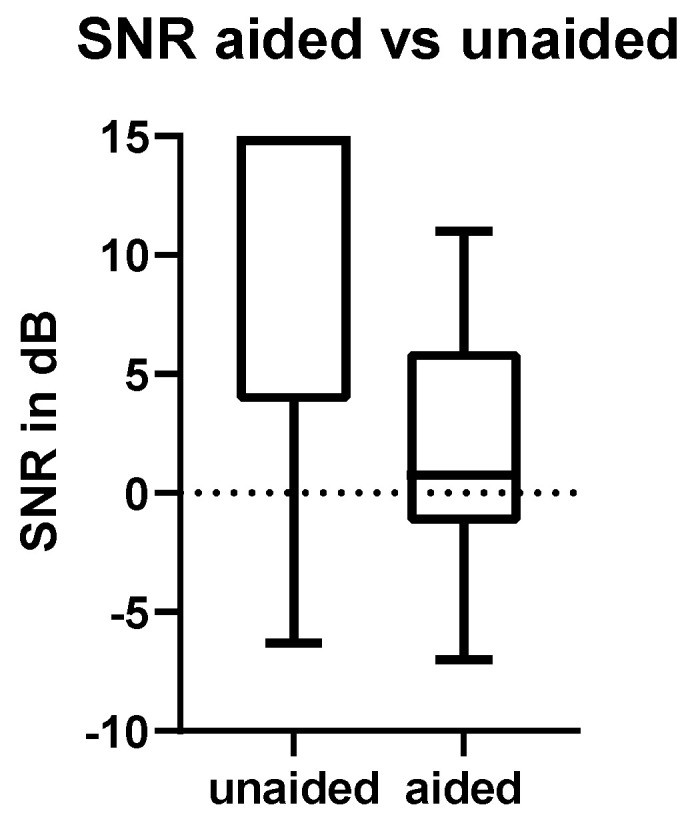
Oldenburger sentence test in noise. Signal-to-noise ratios in free-field with the device turned on (aided) and off (unaided) are shown. The difference is significant: *p* < 0.01.

The average signal-to-noise ratio (SNR) in the Oldenburger sentence test in the unaided condition (where patients did not wear the processor) was 10 dB ± 6.7 dB. Aided, the SNR was 2 dB ± 5.4 dB. There was a significant difference of −8 dB SNR with the device (*p*-value < 0.001) (Figure 4). One patient had to be excluded because of an insufficient knowledge of the German language.

### 3.5. Hearing Implant Sound Quality Index (HISQUI)

Two patients reported >111 points (very good hearing result), one patient had 103 points (good), four patients were between 69 and 88 points, which corresponds to a moderate result, and three patients reported to scored less than 59 points, which corresponds to a poor result. The mean HISQUI was 83 points (moderate).

### 3.6. Association between Audiometric Results and HISQUI 

**Figure 5:**
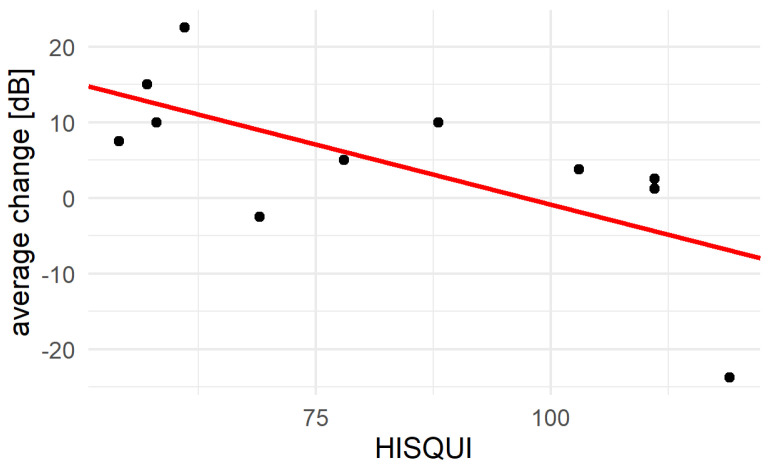
Correlation between change of inner ear function (BC) and the Hearing Implant Sound Quality Index (HISQUI). The individual values are shown as black dots, the correlation is show with the red line.

There was a significant difference between the change of BC and the HISQUI; on average, per dB decrease in BC, the HISQUI decreases by 1.4 points (SE 19.3, *p* = 0.023, adjusted R2 = 0.39) (Figure 5). 

**Figure 6:**
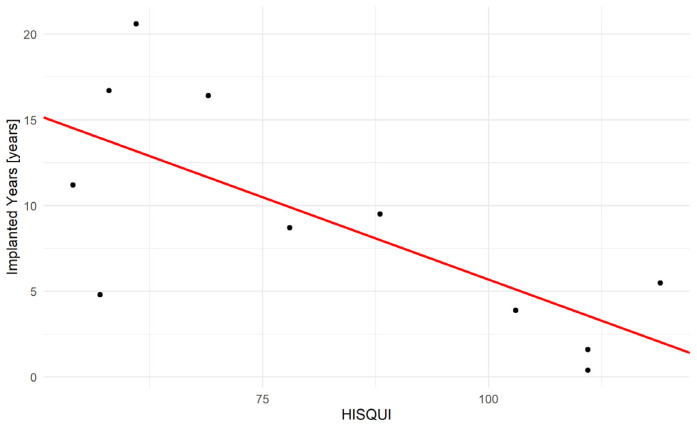
Correlation between years of implantation and the Hearing Implant Sound Quality Index (HISQUI). The individual values are shown as black dots, the correlation is show with the red line.

Additionally, there was a further significant association between the number of years of implantation and the HISQUI. On average, every year after implantation, the HISQUI decreases by 2.7 points (SE 18.17, *p* = 0.013) (see Figure 6). Neither SNR in the aided (*p* = 0.519), nor in the unaided (*p* = 0.459) condition was associated with the HISQUI. 

## 4. Discussion

This study evaluated the long term outcome after VSB implantation. A review of the literature indicates that hearing threshold is not affected in the middle- to long-term follow up after VSB implantation compared to the opposite non-operated ear [8,11]. Various studies have evaluated pre- and postoperative hearing thresholds by pure tone-, free-field-, or speech comprehension audiograms, or determined the effect of surgery on residual hearing. Zahnert et al. [12] reported a significant improvement after VSB implantation in different coupler methods. Audiological performance did not differ significantly between 12 and 36 months after surgery in 24 patients. Grégoire et al. [13] implanted 53 VSB in 46 patients and concluded, in a long term study with a follow up of 2.5 years: Firstly, that the VSB is a safe middle ear implant with no major complications; and secondly, that there was no significant impact on residual hearing. With the implant turned on, the gain in an open field audiogram was 13.9 dB on average. The speech intelligibility with noise was improved by 18.3% on average after two years. Zwartenkot et al. [14] investigated over a mean follow up of 4.4 years the medical and technical outcome, complications, as well as implant survival in 94 patients after the implantation of 128 middle ear implants, 92 of which were VSB implants. In line with other studies, the authors concluded that the VSB is a reliable active middle ear implant for use in sensorineural and mixed hearing loss. The device failure rate was one every 74 years and the revision rate was one every 13 years. A further long term study with 103 patients with a total of 118 VSB implants reported an overall complication rate of 16.1% and a device failure rate of 3.4% over a period of 6.7 years on average. The average implant loss for technical defects was one per 158 years of follow up [15]. The benefit of VSB could be confirmed in our study. The average effective gains with the device turned on in the free-field audiogram were between 23 to 35 dB. Previous studies have reported different hearing gains in patients with mixed or sensorineural hearing loss. Brkic et al. [15] reported smaller functional hearing gains for sensorineural hearing loss of 13.1 dB and higher hearing gains of 20.9 dB for mixed hearing loss. Grégoire et al. [13] reported a mean hearing gain of 19.4 dB between 1000 and 4000 Hz in patients who suffered mostly from sensorineural hearing loss. A systematic review concluded that the VSB provided a gain of 25 to 33 dB HL in sound field audiometry and pure tone averages over different frequencies [16]. Schwab et al. [17] reported hearing gains between 24.2 and 47.5 dB in patients with mixed hearing loss and Spiegel et al. [18] concluded that patients with the broadest air-bone gap of 31.4 dB ± 19.4 dB HL benefitted the most from the Active Middle Ear Implant (AMEI) with a functional gain of 39.0 dB ± 12.8 dB. These results seem to be in line with the hearing gains between 23 and 35 dB in our study, which examined five patients with sensorineural and five patients with mixed hearing loss. However, it has been reported that one of the biggest problems for people who are wearing a hearing aid is understanding in noisy environments [19]. Therefore, speech discrimination in noise was measured. A significant improvement of SNR from 10 dB ± 6.7 dB to 2 dB ± 5.4 dB (gain = 8 dB) was found in our study. This result is in line with previously reported results by Brito et al. [20], who reported a short term improvement from 5.64 (SD, 4.33) to 1.31 (SD, 3.89) (gain = 4.3 dB). Since 2 dB SNR resembles the average normal performance of people without hearing impairments [19], our findings can be considered as a good result. However, even an SNR of 0 dB and below is reported for patients with mild hearing loss results, while an SNR of 5 dB was measured in patients with moderate hearing loss [21]. A direct comparison with other studies is challenging because there is great variety in the type speech test in noise [16]. 

A very important additional factor in determining the outcome of an intervention is patient satisfaction. Since no specific questionnaires for active middle ear implants exist, the HISQUI questionnaire was used because it has proven to be user-friendly and has been used to determine subjective auditory benefit in other implants, such as the Bonebridge hearing implant [7]. Despite good results in audiological tests, only 30% of our patients reported to have a good to very good hearing quality, 40% reported to have a moderate, and 30% reported to have a poor result. Brkic et al. [15] reported that 77% were using the device at the end of the observation period of up to 17.9 years (average 6.7 years). Others found a higher satisfaction rate, although with a shorter follow-up. Mosnier et al. [8] compared audiological performance, satisfaction rate, and side effects of 77 patients who have been using the VSB for five to eight years. They reported a stable satisfaction rating and functional gain provided by the VSB without adverse effects over five or more years. Sterkers et al. [22] reported that 83% of the patients were either satisfied or very satisfied 12 months after surgery. Another study of 20 patients with a mean age of 59 years reported that two years after implantation of a VSB, 65% were satisfied or very satisfied [23]. Our data do not provide insight into whether patient satisfaction decreases over time for any reason. The type of hearing loss is described to affect patient satisfaction after VSB implantation. Higher satisfaction rates was found in mixed and conductive hearing loss compared to sensorineural hearing loss. It was speculated that the different satisfaction levels might be due to different expectations and due to different types of hearing loss with higher gains in conductive hearing loss patients [24]. The lower satisfaction compared to the other studies might be based on the lower percentage of conductive hearing loss patients. However, our study was not designed and powered to further investigate this assumption. 

A direct comparison to other studies is difficult, because various questionnaires are used by different authors. Ihrler et al. used the Glasgow Benefit Inventory (GBI) and compared patients with an active middle ear implant to a matched population with conventional hearing aids [25]. Zwartenkot et al. [26] evaluated long-term outcomes in patient satisfaction using the Abbreviated Profile of Hearing Aid Benefit (APHAB) questionnaire and the Nijmegen Cochlear Implant questionnaire, as well as the GBI. Monini et al., 2017, used the Glasgow Benefit Inventory, the Visual Analog Scale (VAS), and the APHAG to assess the rate of satisfaction in patients with an auditory implant. A further study used a specifically designed survey and the GBI to evaluate patient satisfaction after VSB implantation [22]. Atlas et al. [27] evaluated patients’ satisfaction with the International Outcome Inventory for Hearing Aids and concluded that the scores were significantly higher with the middle ear implant than with conventional hearing aids.

The age of the patient seems to affect speech recognition. Von Gablenz and Holube [28] investigated hearing loss and speech recognition in the elderly. While they reported moderate to good correlations between the audiogram and signal-to-noise ratios, there was a poor correlation between the audiological tests and subjective reported hearing difficulties. Especially people between 60 and 64 years tend to underrate their hearing abilities and overrate their difficulties, while from the age of 70 years onwards, the elderly tend to overrate their hearing abilities and underrate their difficulties. Despite the continuous decline in the pure-tone average and Goettingen sentence test in noise with age > 70, the self-rated hearing ability score stagnated. As shown in Figure 6, there was a correlation between years after implantation and the HISQUI. The longer the implant was used, the lower the subjective benefit seemed to be. With one exception, the patients had a very high HISQUI within five years of implantation. Patients who were using the implants for 10 years and more showed a much lower HISQUI. Even though there are high effective gains in free-field and speech discrimination in noise tests, the patients’ satisfaction seemed to decline. This is in line with another long-term follow up study where the subjective satisfaction decreased at each evaluation. The questionnaire scores dropped by about 10 points over a period of seven years [26]. They speculated that the deterioration over time might be ascribed to advancing hearing loss. Increasing listening difficulties over time are also described for people with conventional hearing aids. Patients <65 years seemed to benefit more than the older patients [29]. 

There was a correlation between the average change of BC threshold (RTA 0.5, 1, 2, 4 in dB) and the HISQUI. The higher the deterioration, the less the subjective hearing implant sound quality. The influence of higher complex central processing that changes with age may be the reason for these findings [28]. 

There are limitations of our study. For a variety of different reasons, we were only able to include 10 out of 40 patients. Therefore, we did not differentiate the subjects into further subgroups such as sensorineural hearing loss vs. mixed hearing loss, and did not differentiate different coupler systems. We did not evaluate individual reasons for study participation, or soft factors such as how many times the patients visited their hearing professionals to adjust the hearing aids. Due to the limited number of included patients, the statistical analysis and interpretation of data need to be interpreted with care. Furthermore, the time of follow-up was variable and ranges from several months to several years. Finally, the HISQUI was only documented once during the follow-up at individual time points.

## 5. Conclusions

Aided thresholds with VSB in free field improved significantly for a follow-up of up to 20 years. Additionally, speech understanding in noise improved significantly. However there was a decay of the subjective sound quality detection (HISQUI) over time, and an increase in BC thresholds. Most likely, the increased BC threshold is due to presbycusis, causing patients to have issues with audibility and therefore decreasing the benefit of VSB. Patients with VSB should be counselled that they may fall out of the indication range with time. Therefore, monitoring of these patients over time should be considered to discuss alternative hearing rehabilitation measures such as cochlear implants if required in good time. However, due to the low number of included patients, investigations on larger patient groups are required to confirm this assumption.

## Figures and Tables

**Table 1:** Patients demographics and characteristics.

Subject	Gender	Age	Follow up (in yrs)	Side	Aetiology
1a *	F	48	1.6	Right	Atresia of the external ear channel with mixed hearing loss
1b *	F	48	0.4	Left	
2	M	71	3.9	Left	Mixed hearing loss after cholesteatoma
3	F	71	9.5	Left	Sensorineural hearing loss
4	M	83	20.6	Left	Sensorineural hearing loss
5	F	54	4.8	Right	Sensorineural hearing loss
6	M	72	8.7	Right	Mixed hearing loss
7	M	62	16.7	Right	Sensorineural hearing loss
8	F	74	11.2	Right	Mixed hearing loss
9	M	49	16.4	Right	Sensorineural hearing loss
10	M	59	5.5	Left	Mixed hearing loss after cholesteatoma

M, male; F, female; * subject 1 was implanted on both sides.

## Data Availability

Data are available upon a reasonable request.
